# Effect of Solvothermal Reaction-Time on Microstructure and Microwave Absorption Properties of Cobalt Ferrite

**DOI:** 10.3390/ma13235331

**Published:** 2020-11-25

**Authors:** Yue Yuan, Shicheng Wei, Yi Liang, Bo Wang, Yujiang Wang

**Affiliations:** National Key Laboratory for Remanufacturing, Army Academy of Armored Forces, Beijing 100072, China; yyyue11111@163.com (Y.Y.); liangyi365@126.com (Y.L.); wangbobo421@163.com (B.W.); hitwyj@126.com (Y.W.)

**Keywords:** cobalt ferrite, reaction time, structure, microwave absorption properties

## Abstract

Cobalt ferrite is synthesized via a simple solvothermal method. Then, the effect of the degree of cobalt-ferrite growth on its morphology, structure, electromagnetic performance, and microwave absorption is studied as a function of the solvothermal reaction time. When the reaction time during synthesis is 8 h, the structure of cobalt ferrite is hollow spheres. In addition, when the reaction time is 12 h and 16 h, it becomes a submicron sphere with a diameter of 100–150 nm. With the increase of reaction time, cobalt ferrite underwent the process of cobalt ferrite formation, hollow structure formation, hollow structure disappearance, agglomeration separation and reagglomeration in 4–16 h. In general, CoFe_2_O_4_-8h shows better microwave absorption-the effective absorption bandwidth is 9.84 GHz (6–15.84 GHz) for a thickness of 1.72–3.72 mm. This represents a minimum return loss of −47.24 dB. A better understanding of both the synthesis parameters and the relationship between structure and electromagnetic properties can open new possibilities for applications and the development of microwave absorbing materials.

## 1. Introduction

Ferrites are ferromagnetic metal-oxides with both magnetic and electric absorption capabilities [1,2]. Because of its stable chemical properties, strong high-frequency permeability, wide frequency-band and strong absorption, ferrites are often used as microwave absorbers [3,4,5]. Based on their crystal structure, ferrite materials are mainly spinel-type, magnetoplumbite-type, and garnet-type [6].

Cobalt ferrite, in particular, is one of the most widely studied spinel-type ferrites due to its high saturation magnetization and high-frequency permeability, high Snoek-limit, excellent chemical stability, high mechanical hardness, high cubic magneto-crystalline anisotropy, and low cost [7,8,9]. It is widely used as an electromagnetic absorber. However, single-component cobalt ferrite usually has high density, poor impedance-matching, narrow absorption frequency band, and low absorption [10]. Therefore, processes such as hollowing, core-shell, nano, fiber, and creating composites can effectively reduce the density, tune the electromagnetic parameters, increase the absorption strength, and broaden the absorption band [11,12,13,14]. For example, Wang et al. [15] used cobalt ferrite to coat hollow glass-microspheres and a conductive polymer PPY to form a three-layer sandwich-structure material. When the thickness of this material was 2.58 mm, the effective absorption range covered the entire X-band. Zhang et al. [7] prepared a hollow spherical cobalt ferrite and carbon nanotube composite material with a core-shell structure in situ using chemical vapor deposition. The minimum return-loss was −32.8 dB and the effective bandwidth was 5.7 GHz.

In this paper, the effect of reaction time on the phase and structure of cobalt ferrite was studied in depth, and the effect of cobalt ferrite structure on electromagnetic parameters, microwave absorption mechanism and properties analyzed intensively. This study has some guiding significance for the synthesis of cobalt ferrite and cobalt ferrite composite absorbing materials.

## 2. Experimental Procedures

### 2.1. Sample Preparation

13.98 g ferric chloride hexahydrate (Tongguang Fine Chemical Co. LTD, Beijing, China) and 7.14 g cobalt chloride hexahydrate (Aladdin Reagent Co. LTD, Shanghai, China) were added to 400 mL of ethylene glycol (Fuyu Fine Chemical Co. LTD, Tianjin, China), mechanically stirred until there no large particles could be observed. Then, 36 g urea (Guangfu Technology Development Co., LTD, Tianjin, China) and 40 g polyvinylpyrrolidone (Aladdin Reagent Co. LTD, Shanghai, China) were added to the above mixture, and after mechanically stirred for 0.5 h, and subsequently transferred into a Teflon-lined stainless-steel autoclave (500 mL capacity and PTFE lined, Zhuoran Instrument equipment Co. LTD, Henan, China) and kept at 200 °C for different time (4 h, 8 h, 12 h, 16 h). After cooling to room temperature, the product was washed with deionized water and anhydrous ethanol (Beijing Chemical Works, Beijing, China) several times, and dried in a vacuum drying oven (2ZX-2, Yiheng Scientific Instrument Co. LTD, Shanghai, China) at 60 °C for 24 h.

### 2.2. Characterization and Measurement

The surface morphology of the samples was analyzed using a ZEISS Sigma 500 field-emission scanning electron microscope (FEI-SEM, ZEISS Sigma 500, Carl Zeiss AG, Jena, Germany, the samples were demagnetized and dispersed in ethanol, and ultrasound for 30 min) and a JEOL JEM-2100F transmission electron microscope (TEM, JEOL JEM-2100F, JEOL, Tokyo, Japan, the samples were dispersed in ethanol, and ultrasound for 30 min, then prepared with ordinary carbon grid). The phase analysis of the cobalt ferrite products was conducted with a UltimalV X-ray diffraction analyzer (XRD, UltimalV, Rigaku, Tokyo, Japan) from 20° to 80°, with a scanning rate of 2°/min, using Cu Kα (1.54 Å). A PNA-N5244A vector network analyzer (VNA) was used to test the electromagnetic parameters of the samples, and the sample was mixed with paraffin using a mass ratio of 6:4 to create a ring sample with an outer diameter of 7 mm, an inner diameter of 3 mm, and a height of 2–3 mm. The magnetic properties of the samples were measured with a physical property measurement system (PPMS, Quantum Design PPMS-9, Quantum Design, San Diego, CA, USA) in the field of ±10 kOe at room temperature.

## 3. Results and Discussion

### Microstructure

**Figure 1:**
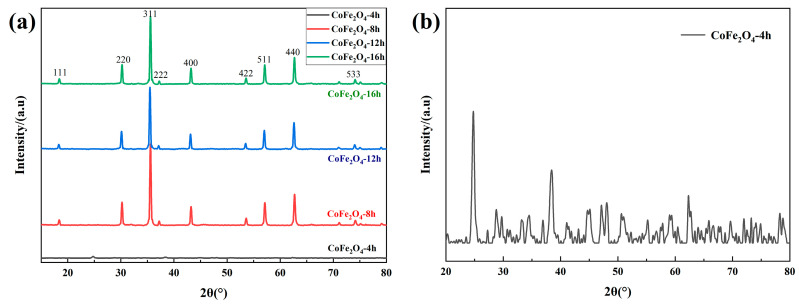
X-ray diffraction analyzer (XRD) pattern of cobalt ferrites; (**a**) the cobalt ferrite with different reaction time; (**b**) the product of 4h reaction time.

Figure 1a shows the XRD pattern of cobalt ferrite for different reaction times. Combined with JCPDS File No 22-1086, characteristic peaks appeared at 18.5°, 30.1°, 35.5°, 43.1°, 53.5°, 60°, 62.6°, and 74.1°, which correspond to the crystal plane of CoFe_2_O_4_. As shown in Figure 1b, when the reaction time was 4 h, there are some characteristic peaks of lower intensity. By comparing with the standard spectrum, they are mixed substances of ferric tetroxide and organic compounds containing cobalt and iron. When the reaction time increased, the diffraction pattern did not change much, which indicates that the increase of the reaction time has little effect on the phase and crystallinity of cobalt ferrite.

**Figure 2:**
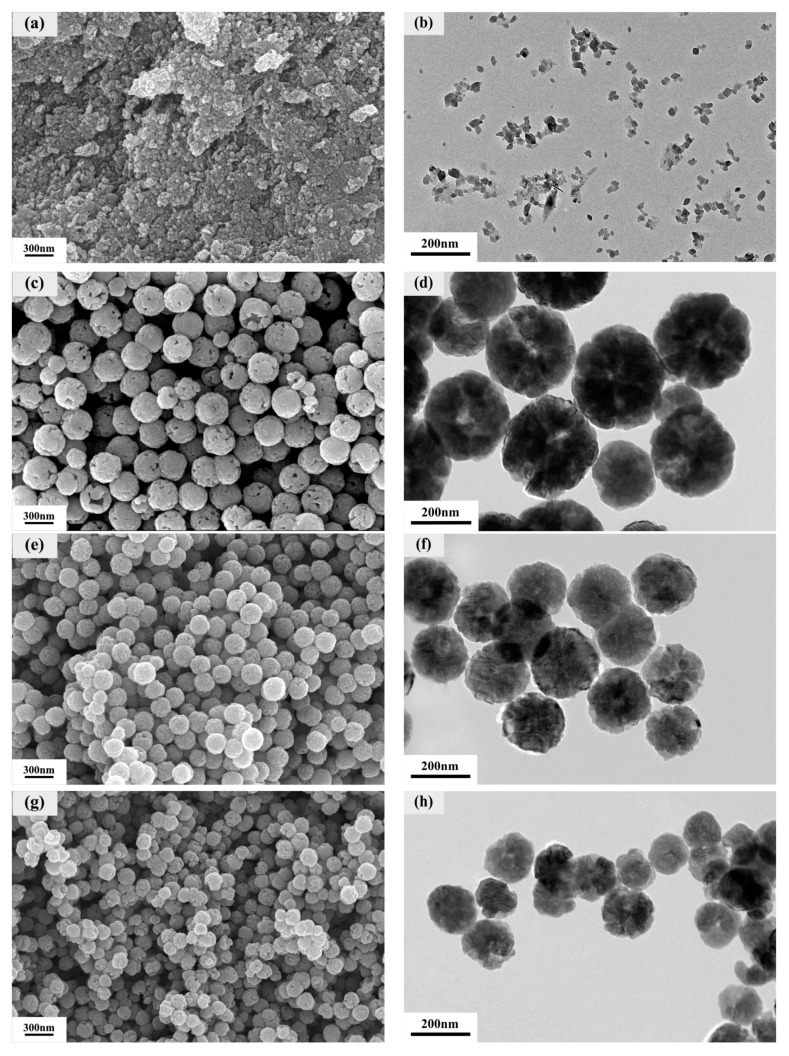
Scanning electron microscope (SEM) and transmission electron microscope (TEM) images of cobalt ferrite for different reaction times. SEM images: (**a**) 4 h, (**c**) 8 h, (**e**) 12 h, (**g**) 16 h; TEM images: (**b**) 4 h, (**d**) 8 h, (**f**) 12 h, (**h**) 16 h.

To analyze the effect of reaction time on the growth and synthesis of cobalt ferrite, the samples were investigated using a scanning electron microscope and a transmission electron microscope. The results are shown in the figures. When the reaction time is 4 h, the product has the form of broken particles, with larger aggregates, which cannot be distinguished clearly. Many cobalt ferrite nanoparticles formed. When the reaction time increased, smaller nanoparticles cluster together to form larger particles with reduced surface energy [16]. Therefore, it can be seen from Figure 2c,d that the cobalt ferrite sphere consists of large numbers of cobalt-ferrite crystal grains, with a particle size of about 250 nm. In Figure 2d, the edge color of the small sphere is darker and the center color is lighter. In conjunction with the internal situation of the broken ball shown in Figure 2c, it can be concluded that the cobalt ferrite reacted for 8 h and formed a hollow structure. The reason for this is mainly related to gas-assisted Ostwald-ripening. On the one hand, a certain amount of carbon dioxide gas is generated by the thermal decomposition of the reaction reagent urea, which provides a template for the synthesis of cobalt-ferrite hollow spheres. On the other hand, because of Ostwald-ripening, the surface energy of the internal grains, which were formed during the early stage, is higher [17]. As the reaction time increases, the grains evaporate, dissolve, and attach to the surface of the recently formed grains. Finally, a hollow structure is formed via repeated sedimentation and maturation.

For a reaction time of 12 h, the hollow structure of the cobalt ferrite almost disappears in the figure, and the product is a solid pellet of uniform size, with a particle diameter of about 150–200 nm. Now, the cobalt ferrite pellet shows good dispersion. As the reaction time increases, the originally formed hollow cobalt-ferrite spheres are broken. However, due to the principle of minimum energy, the particles of the broken cobalt-ferrite reassemble to form compact solid spheres. When the reaction time is increased to 16 h, Figure 2g,h shows that the surface of the cobalt ferrite spheres is broken, and they continue to decrease in size, reaching a particle size of about 100–150 nm.

This is because, under the effect of high temperature and the repulsion of the electric and magnetic field between the particles, the agglomerates are separated, reducing the particle size of cobalt ferrites. In addition, the cobalt-ferrite pellets failed to completely form broken particles mainly due to the high concentration of PVP in the reaction system. The mechanical inhibition of PVP can restrain the growth of cobalt-ferrite particles, which causes broken particles. The small particles continue to self-assemble into complete cobalt-ferrite particles.

As a summary, the reaction time has a great effect on the growth of cobalt ferrite, and the cobalt ferrite undergoes the process of forming cobalt ferrite, forming hollow spheres, hollow structure damaging, aggregates dispersing and agglomerating again in 4–16 h.

Cobalt ferrite is a kind of microwave absorbing material with both electric and magnetic loss capacity. Therefore, the permittivity and electric loss mechanism of cobalt ferrite with different reaction times have been studied.

Generally, the various polarization relaxations caused by the polarization of electrons and molecules in a material cannot keep up with the changes in the external high-frequency electric field, resulting in the permittivity becoming a plural, and the formula is as follows:

(1) $$\varepsilon =\varepsilon^{\prime }-i\varepsilon^{\prime \prime }.$$

For dielectric materials, the permittivity is a constant related to frequency, which reflects the relationship between the alternating electric field strength (*E*) and electric displacement vector (*D*), and the formula is:

(2) $$D=\varepsilon_{0}E+P$$

(3) $$\varepsilon^{\prime }=\frac{D_{0}}{E_{0}}cos\delta$$

(4) $$\varepsilon^{\prime \prime }=\frac{D_{0}}{E_{0}}sin\delta ,$$

where *ε*_0_ is the permittivity in a vacuum, *P* is the intensity of polarization, *ε*′ and *ε*″ are the real and imaginary part of permittivity. *δ* is the phase angle of *D* and *P* behind the alternating electric field. The polarization of the dielectric can be expressed by the permittivity.

**Figure 3:**
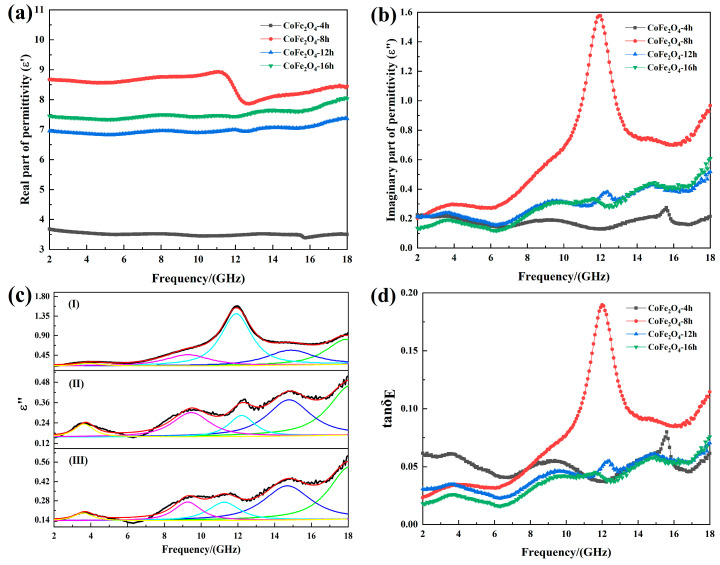
(**a**) Real part of the effective permittivity, (**b**) imaginary part of the effective permittivity, (**c**) the fitting curve of the imaginary part of effective permittivity, (I) 8 h; (II) 12 h; (III) 16 h; (**d**) the dielectric loss factor.

Figure 3 shows the real and imaginary parts of the effective permittivity and the dielectric loss factor of cobalt ferrite for different reaction times. By comparing the effective permittivity of the four sets of samples, it is found that there are two types in the 2 GHz–18 GHz band: one is a smooth curve of 4 h, 12 h and 16 h cobalt ferrite, and the other is a curve of 8 h cobalt ferrite with a prominent peak. This is because when the frequency of the applied electric field is too high, the polarization frequency of the charge inside the material cannot keep up with the frequency of the electric field. As a result, polarization relaxation occurs. Polarization relaxation usually causes the real part of the permittivity to fluctuate [18].

As shown in Figure 3a,b, when the reaction time is 4 h, the CoFe_2_O_4_-4h has a low effective permittivity, because the cobalt ferrite particles are not completely formed. In addition, the permittivity is not only related to the phase of the material, but also related to the microstructure. When the reaction time increases to 8h, the effective permittivity is the largest. This is because when the cobalt ferrite fills the paraffin-based powder with the same mass, the hollow cobalt ferrite occupies a larger volume, resulting in a greater probability of contact between particles, which causes the interface polarization to increase. On the other hand, it is easy to form a conductive network, according to free-electron theory (*ε*″ = 1/2*ε_0_πρf*, where *ρ* is the resistivity.), the imaginary part of the permittivity is proportional to the conductivity of the material [19]. Therefore, the cobalt ferrite with a hollow structure has a large real part of effective permittivity.

In order to analyze the electrical loss mechanism of cobalt ferrite deeply, the Landau-Lifshitz equation is introduced to fit the imaginary part of permittivity, as shown as follows [20]:

(5) $$\varepsilon^{\prime }=\varepsilon_{\infty }+\frac{\left(\varepsilon_{0}-\varepsilon_{\infty }\right)\left[1-{\left(f/f_{r}\right)}^{2}\right]}{{\left[1-{\left(f/f_{r}\right)}^{2}\right]}^{2}+{\left(f/f_{d}\right)}^{2}}$$

(6) $$\varepsilon^{\prime \prime }=\frac{\left(\varepsilon_{\infty }-\varepsilon_{0}\right)\left(f/f_{d}\right)}{{\left[1-{\left(f/f_{r}\right)}^{2}\right]}^{2}+{\left(f/f_{d}\right)}^{2}}$$

where *ε*_0_ and *ε*_∞_ are static and limit permittivity. *f_r_* and *f_d_* are natural resonance frequency and Debye frequency respectively.

Figure 3 c is the fitting curve of the imaginary part of effective permittivity. It can be seen from Figure 3c that the curves of the imaginary part of effective permittivity of the three cobalt ferrites have five fitted resonance peaks, and the fitted frequency and the actual frequency are shown in Table 1. After contrast, the frequency obtained by the fitting is basically consistent with the actual frequency.

The cobalt ferrite prepared in this experiment has multiple dielectric resonances in the frequency range of 2–18 GHz. Compared with the other two sets of samples, the peak of CoFe_2_O_4_-8h is significantly larger. In the frequency range of 2–18 GHz, the dielectric polarization of cobalt ferrite mainly comes from interface polarization, and the hollow structure mainly enhances the interface polarization relaxation ability of the material, which causes the imaginary part of the effective permittivity to become larger.

When the reaction time is 12 h and 16 h, the cobalt ferrites are a solid spherical structure, and the real part of effective permittivity of CoFe_2_O_4_-16h is greater than that of CoFe_2_O_4_-12h, because the particle size of CoFe_2_O_4_-16h is smaller, even reaching the nanometer scale. The decrease in particle size will result in a large increase in particle defects, dipoles, and surface dangling bonds, which enhance the dielectric polarization ability, thus making the effective permittivity higher.

The dielectric loss factor (*tanδ_E_*) can be used to evaluate the electric loss capacity of the absorbing material [21].

(7) $$tan\delta_{E}=\frac{\varepsilon^{\prime \prime }}{\varepsilon^{\prime }}.$$

As shown in Figure 3d, when the reaction time is 8 h, the cobalt ferrite has a strong electric loss ability. However, the tanδ_E_ of CoFe_2_O_4_-12h and CoFe_2_O_4_-16h is lower than CoFe_2_O_4_-4h, due to the large real parts and low imaginary parts of effective permittivity of CoFe_2_O_4_-12h and CoFe_2_O_4_-16h. In summary, when the reaction time is 8h, the cobalt ferrite has strong dielectric polarization ability and electric loss ability.

As a ferromagnetic material, electromagnetic energy can be converted into other energy in cobalt ferrite due to its unique magnetic loss mechanism, so it is necessary to research the magnetic properties. The relative permeability in a changing magnetic field is expressed as [22]:

(8) $$\mu_{r}=\frac{B_{m}}{\mu_{0}H_{m}}e^{-j\theta }.$$

*Bm* is the magnetic induction, *Hm* is the magnetic field, and *θ* is the phase angle of *B* is lagging *H*. From this, it can be deduced that in a dynamic magnetic field, the permeability is a complex number, which can be divided into two parts. It can be formulated as follows:

(9) $$\mu_{r}{}^{\prime }=\frac{B_{m}}{\mu_{0}H_{m}}cos\theta$$

(10) $$\mu_{r}{}^{\prime \prime }=\frac{B_{m}}{\mu_{0}H_{m}}sin\theta .$$

The relationship between the magnetic field and the magnetic induction is:

(11) $$M=\chi H=\frac{\chi }{1+\chi }\frac{B}{\mu_{0}}$$

(12) $$B=\left(H+M\right)\mu_{0}.$$

After substitution, the following relationship between permeability and magnetization can be obtained:

(13) $$\mu_{r}{}^{\prime }=(1+\frac{M}{H})cos\theta$$

(14) $$\mu_{r}{}^{\prime \prime }=(1+\frac{M}{H})sin\theta .$$

**Figure 4:**
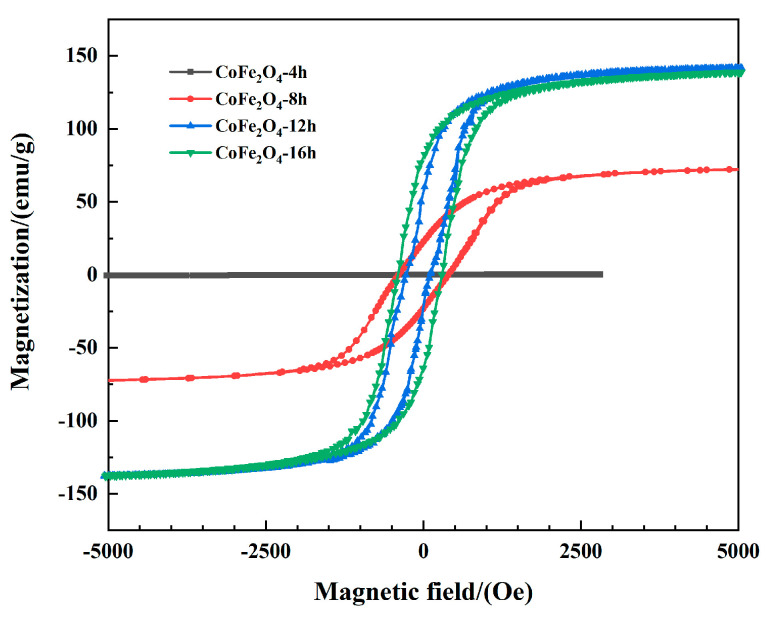
Hysteresis loops of CoFe_2_O_4_ measured at room temperature.

To analyze the effect of the different reaction times of cobalt ferrites on the magnetic properties, four groups of samples were tested using a comprehensive physical property-measurement system at room temperature. In addition, the *M-H* curve was recorded. The saturation magnetization and coercivity of the material can be observed via the magnetization curve. Figure 4 shows that the magnetization curves of the cobalt ferrites (with reaction times of 8 h, 12 h and 16 h) show a hysteresis loop pattern, which suggests the formation of ferromagnetic cobalt ferrite. When the reaction time was 4 h, and because it occurred in the early stage of cobalt-ferrite growth, the product had almost no magnetic properties. When the reaction time increases, the cobalt ferrite particles grow mature and exhibit excellent magnetic properties. By comparing the hysteresis loops of cobalt ferrite at 8 h, 12 h, and 16 h, it can be found that the degree of growth of cobalt ferrite has a great effect on the magnetic properties. When the reaction time is 8 h, a hollow cobalt ferrite formed. For the magnetic properties, the coercive force is closely related to the grain size. For generally spherical particles, the coercive force increases as the particle size decrease. After the particles reach the critical single domain size, their coercive force decreases with decreasing size. For hollow spheres, when the size of the hollow reaches a certain value, and the shell thickness reaches the critical single domain size, the hollow sphere can assume an approximate single-domain structure. Compared with the multi-domain structure, the magnetization process transforms from domain wall motion to magnetic moment rotation, and its coercive force is greater. The coercive force is inversely proportional to the saturation magnetization. The saturation magnetization of the CoFe_2_O_4_-8h is smaller than for the other two samples. When the reaction time is 12 h, due to the formation of solid cobalt-ferrite balls, the advantage of the hollow is lost. As a result, the coercive force decreases and the saturation magnetization increases. When the reaction time is 16 h, the size of the cobalt–ferrite particles decreases but it is larger than the critical single-domain size. Therefore, when the particle size decreases, the coercive force increases, and the saturation-magnetization decreases. In conclusion, the saturation magnetization and magnetic properties of little size cobalt ferrites are higher than those of hollow cobalt ferrites.

**Figure 5:**
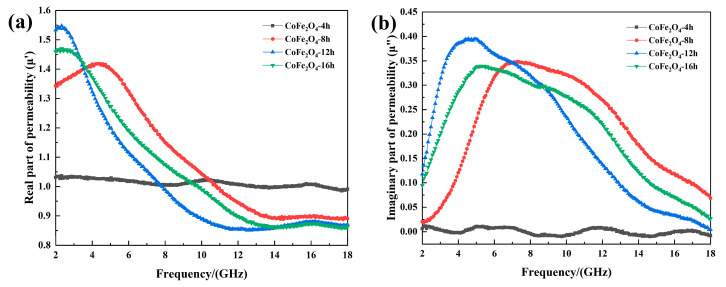
(**a**) The permeability of cobalt ferrite; (**b**) imaginary part of the permeability.

Figure 5 shows the real and imaginary part of effective permeability and magnetic loss factor of cobalt ferrite with different reaction time. As shown in Figure 5a,b, When the reaction time is 4 h, the cobalt ferrite crystal structure is unstable due to its in the early stage of growth. This means that it cannot show excellent effective permeability as it at high frequencies. In addition, when the reaction time is 8 h, 12 h and 16 h, as the frequency increases, the real part of effective permeability first rises to the maximum value, and then decreases rapidly, the final value stabilizes at about 0.9. The real part of the effective permeability of the three samples has a resonance peak near 4.4 GHz, 2.32 GHz and 2.29 GHz. Meanwhile, in the frequency range of 2–18 GHz, the imaginary part of the effective permeability of the three sets of samples has a wide resonance band.

**Figure 6:**
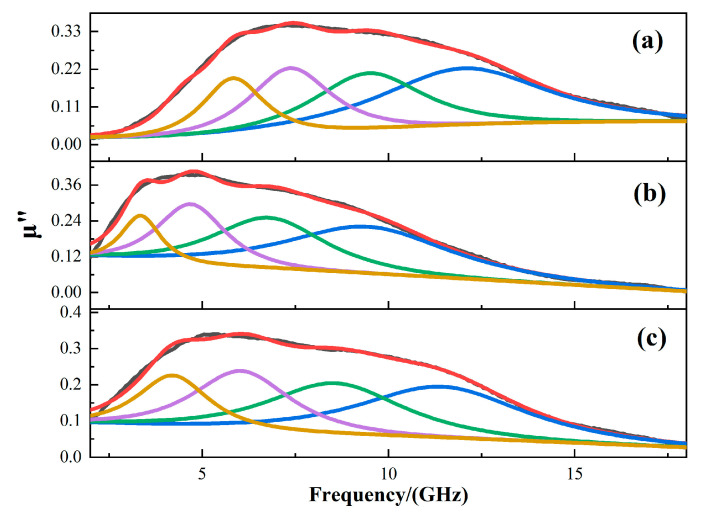
The fitting curve of the imaginary part of effective permeability, (**a**) 8 h; (**b**) 12 h; (**c**) 16 h.

Generally, the magnetic loss mechanism of ferrite in the range of 2–18 GHz mainly includes natural resonance loss and exchange resonance loss [23]. In order to analyze the magnetic loss mechanism of cobalt ferrite deeply, the Landau–Lifshitz equation is introduced, as shown as follows:

(15) $$\mu^{\prime }=B+\overset{n}{\underset{i=1}{\sum }}x_{0}\frac{1-{\left(\frac{f}{f_{ri}}\right)}^{2}\left(1-\alpha_{i}^{2}\right)}{{\left[1-{\left(\frac{f}{f_{ri}}\right)}^{2}\left(1+\alpha_{i}^{2}\right)\right]}^{2}+4\alpha_{i}^{2}{\left(f/f_{ri}\right)}^{2}}$$

(16) $$\mu^{\prime \prime }=\overset{n}{\underset{i=1}{\sum }}x_{0}\frac{\left(\frac{f}{f_{ri}}\right)\alpha_{i}\left[1+{\left(\frac{f}{f_{ri}}\right)}^{2}\left(1+\alpha_{i}^{2}\right)\right]}{{\left[1-{\left(\frac{f}{f_{ri}}\right)}^{2}\left(1+\alpha_{i}^{2}\right)\right]}^{2}+4\alpha_{i}^{2}{\left(f/f_{ri}\right)}^{2}},$$

where *f* is frequency, *f_ri_* is the resonance frequency, *α_i_* is the damping coefficient, and *X*_0_ is the intensity of the band. Because the imaginary part of the permeability has a wide resonance peak, we use the Equation (16) to fit the permeability, and the fitting results are shown in Figure 6. The natural resonance frequency of cobalt ferrite can be calculated by Kittel formula (*f_ri_* = (*γ*_0_/2*π*) × *H_α_*)). Where *γ_0_* is the gyromagnetic ratio; *H_α_* is the anisotropy field.

As illustrated in Figure 6, the fitting imaginary part of the effective permeability is almost the same as the measured one, and each of them can be fitted by four peaks. The fitting parameters are shown in Table 2. The four peaks correspond to the natural resonance of iron atom, cobalt ferrite and ferric oxide, and the peak at high frequency is the exchange resonance peak. It can be seen that as the reaction time increases, the frequency of the resonance peak decreases first and then increases, indicating that both hollow structures and small-sized structures can shift the resonance peak to high frequencies.

For most spherical magnetic materials, the two dispersion characteristic parameters- resonance frequency and static permeability, can be determined according to the Snoek formula [24]:

(17) $$\left(\mu_{i}-1\right)f_{0}=\frac{2\gamma M_{S}}{3\pi }$$

In Equation (17), *μ_i_* is static permeability, *f*_0_ is the resonance frequency, *γ* is the magnetogyric ratio and *M_s_* is the saturation magnetization. It can be concluded that the resonance frequency is inversely proportional to the static permeability and their numerical values are limited by the saturation magnetization.

By comparing the fitting imaginary parts of the effective permeability of the CoFe_2_O_4_-8h, CoFe_2_O_4_-12h and CoFe_2_O_4_-16h, the resonance frequency of CoFe_2_O_4_-8h and the effective permeability of CoFe_2_O_4_-16h are improved. It can be concluded that when the cobalt ferrite is small in size or hollow in structure, the Snoek’s limit of the material can be effectively increased and the permeability at higher frequencies can be improved.

The Magnetic loss factor (*tanδ_M_*) can be used to evaluate the magnetic loss capacity of the absorbing material [21].

(18) $$tan\delta_{E}=\frac{\mu^{\prime \prime }}{\mu^{\prime }}.$$

**Figure 7:**
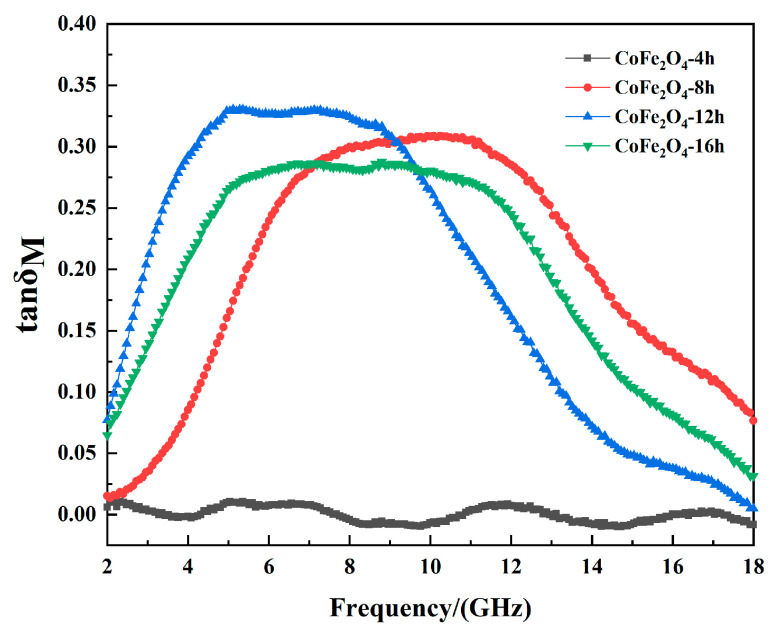
The magnetic loss factor of cobalt ferrite.

Figure 7 is the magnetic loss factor of cobalt ferrite with different reaction times. As the reaction time increases, the magnetic loss band of cobalt ferrite first moves to high frequency and then to low frequency, which corresponds to the analysis of resonance peaks.

For microwave absorbing materials, impedance characteristic and attenuation coefficient are the main factors affecting the absorbing properties. The characteristic impedance *Z* of the material and the wave impedance *Z*_0_ outside the material are important factors that determine the impedance matching characteristics. When the electromagnetic wave propagates to the surface of the material, its reflection coefficient is *R*. The expression for this is as follows [25]:

(19) $$Z=Z_{0}\sqrt{\mu_{1}/\varepsilon_{1}\;}$$

(20) $$Z_{0}=\sqrt{\mu_{0}/\varepsilon_{0}}$$

(21) $$R=\frac{Z_{1}-Z_{0}}{Z_{1}+Z_{0}}.$$

Here, *μ*_1_, *μ*_0_, and *ε*_1_, *ε*_0_ are the permeability and permittivity of the material and vacuum. When Z and Z_0_ are equal, the reflection coefficient is 0, and all electromagnetic waves enter the material. This is called impedance matching.

The electromagnetic attenuation coefficient *α* is an important indicator to measure the attenuation capability of incident electromagnetic waves of the material. It is mainly determined by the real and imaginary parts of the permittivity and permeability. Its value can be calculated using the following formula [26]:

(22) $$\alpha =\frac{\sqrt{2}\pi f}{c}\sqrt{\mu^{\prime \prime }\varepsilon^{\prime \prime }-\mu^{\prime }\varepsilon^{\prime }+\sqrt{{{(\mu }^{\prime \prime }\varepsilon^{\prime \prime }-\mu^{\prime }\varepsilon^{\prime })}^{2}+(\varepsilon^{\prime }\mu^{\prime \prime }+\varepsilon^{\prime \prime }\mu^{\prime }{)}^{2}}}.$$

**Figure 8:**
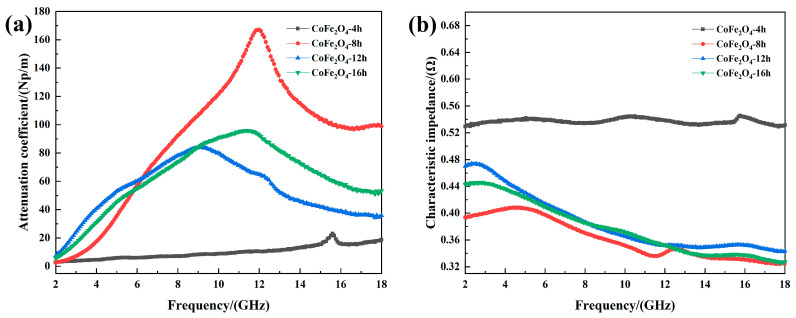
(**a**) Attenuation coefficient and (**b**) characteristic impedance of cobalt ferrite.

Figure 8 shows the attenuation coefficient and characteristic impedance of cobalt ferrite with different reaction times. Figure 8a shows that the attenuation coefficient of CoFe_2_O_4_-8h is better, and the peak frequency is higher. Furthermore, CoFe_2_O_4_-8h has a larger effective permittivity, which results in a larger attenuation coefficient because the frequency of its effective permittivity and effective permeability peak are higher than for the other three groups of cobalt ferrites, and the CoFe_2_O_4_-8h shows stronger electromagnetic wave attenuation at high frequencies. The special hollow structure of cobalt ferrite leads to multiple reflections of electromagnetic wave energy within its cavity. The hollow structure effectively improves conduction loss, which means the attenuation coefficient of CoFe_2_O_4_-8h is higher than for CoFe_2_O_4_-12h and CoFe_2_O_4_-16h. In addition, the attenuation coefficient of CoFe_2_O_4_-16h is slightly higher than for CoFe_2_O_4_-12h. This is the result of an increase in specific surface area, which leads to a higher degree of surface polarization relaxation and the magnetic crystal anisotropy coefficient. However, electromagnetic wave absorption depends on the attenuation coefficient and impedance matching characteristics. The increase of the effective permittivity can result in a larger ratio of effective permittivity to effective permeability, which leads to a smaller characteristic impedance of the material. As shown in Figure 8b, the characteristic impedance of CoFe_2_O_4_-8h is smaller than for the other three groups. It can be found that during the preparation of the absorption material, the electromagnetic parameters should be adjusted correctly. Furthermore, the attenuation ability and impedance matching characteristics of the material should be considered.

In order to visually describe the effect of reaction time on the microwave absorption performance, a three-dimensional model (return-loss/frequency/thickness) was established according to the transmission line theory [27,28]:

(23) $$R_{L}=20log\left|\frac{Z_{in}-Z_{0}}{Z_{in}+Z_{0}}\right|$$

(24) $$Z_{in}=Z_{0}\sqrt{\mu_{r}/\varepsilon_{r}\;}tan\;h\left(j\frac{2fd\pi }{c}\sqrt{\mu_{r}\varepsilon_{r}}\right).$$

In the equation, *h* represents the Planck constant, *c* represents the velocity of the electromagnetic wave in vacuum, *f* is the frequency, *d* is the thickness of the material, *Z_in_* is the input impedance, *Z*_0_ is the characteristic impedance of electromagnetic wave in free space, *μ*_0_ and *ε*_0_ are the permeability and permittivity of free space, *ε_r_* and *μ_r_* are the permittivity and permittivity of the material.

**Figure 9:**
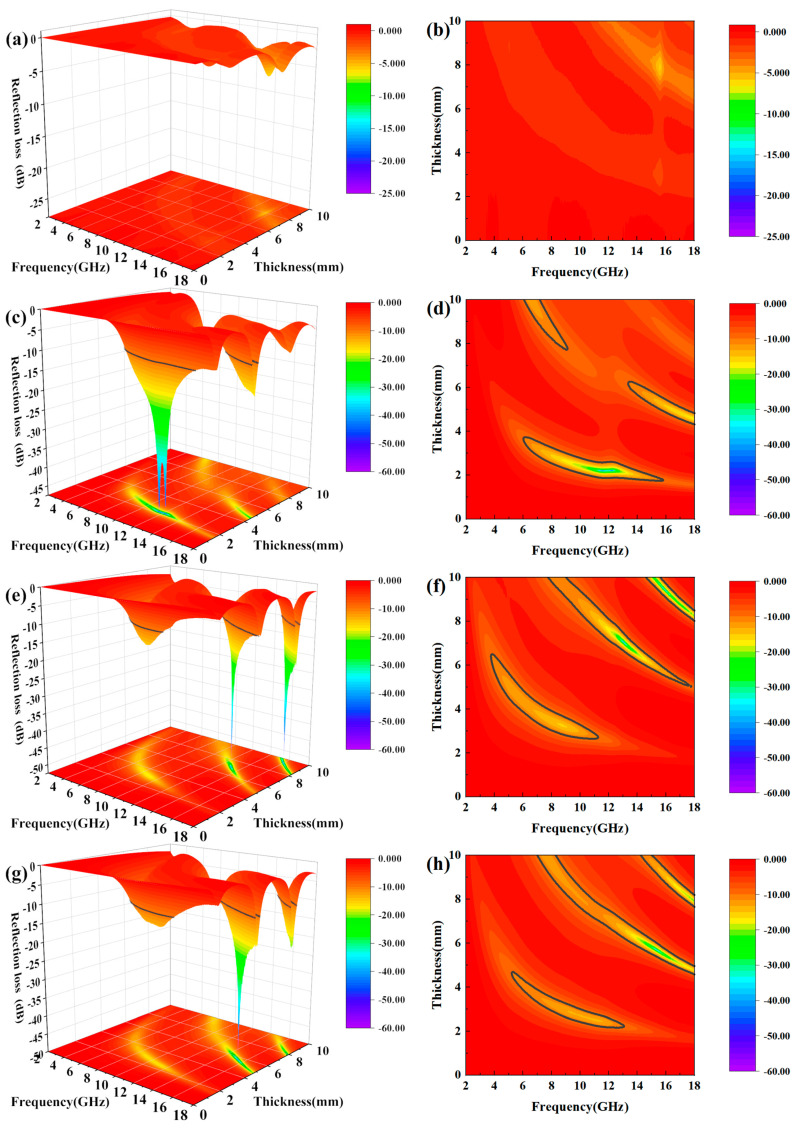
(**a**,**c**,**e**,**g**) Three-dimensional and (**b**,**d**,**f**,**h**) contour-map of reflection loss.

Figure 9 shows the three-dimensional images (return-loss/frequency/thickness) of cobalt ferrite for different reaction times. As the reaction time increases, the effective absorption band at low thickness shifts first towards low frequencies and then to the high frequencies. This is identical to the trend of the attenuation coefficient mentioned above. When the reaction time is 4 h, the electromagnetic wave attenuation ability of cobalt ferrite is very weak. In addition, there is no effective absorption band. In the low thickness range (*d* < 6 mm), when the reaction time is 8 h and the thickness is 1.72–3.72 mm, the effective absorption bandwidth is 9.84 GHz (6–15.84 GHz), with a minimum return-loss of −47.24 dB. When the reaction time is 12 h, at the same thickness, the effective absorption bandwidth is 4.96 GHz (6.32–11.28 GHz), with a minimum return loss of −15.27 dB. When the response time is 16 h, the effective absorption bandwidth is 6.8 GHz (6.24–13.04 GHz), with a minimum return loss of −13.59 dB. When the reaction time is 8 h, the cobalt ferrite shows better absorption for low material thickness.

In summary, the absorbing mechanisms of cobalt ferrite are mainly conduction loss, dielectric relaxation loss, natural resonance loss and exchange resonance loss. When the reaction time is 8 h, the cobalt ferrite has a hollow structure. When electromagnetic waves enter the cavity of the material, it can form multiple reflections, thus increasing the loss of electromagnetic wave energy [29]. When the size of cobalt ferrite particles is reduced to the nanometer scale, due to its small size effect, the specific surface area of the particles is significantly larger, and the number of surface dangling bonds increase, which leads to higher polarization relaxation loss. Therefore, both the small-sized structure and the hollow structure can improve the absorbing performance of the material.

When the electromagnetic wave propagates to the absorbing layer, part of the electromagnetic wave (*W*_1_) is directly reflected due to impedance mismatch, while the rest enters the absorbing layer. When it reaches the metal backing, a second reflection occurs. The mechanism for the electromagnetic-wave loss in the absorbing material is mainly the combination of electromagnetic attenuation of the absorbing layer and the interference cancellation of *W*_1_ and *W*_2_.

In general, the absorption of the material mainly depends on the matching thickness and the electromagnetic parameters of the material. To make the two electromagnetic waves interfere completely, their phase difference must be an odd multiple of 1/2 wavelength. Because the electromagnetic waves propagate twice within the absorbing layer, if the layer thickness is equal to 1/4 wavelength, it can lead to complete interference cancellation. This can be expressed as follows [30]:

(25) $$t=\frac{nc}{4f\sqrt{\left|\mu_{r}\right|\left|\varepsilon_{r}\right|}}\;n=1,3,5,\ldots$$

Here, *t* is the material thickness that satisfies the 1/4 wavelength model, *c* is the speed of light, and *f* is the frequency of the electromagnetic waves. It can be concluded that if *f* is a known quantity, the matching thickness t only depends on the modulus of the permeability and the permittivity (*|μ_r_||ε_r_|*). Therefore, *|μ_r_||ε_r_|* should be enhanced to obtain a thinner absorbing material.

As a result of our analysis, a material’s absorption can be improved by any of the following: (1) increase of the product of permittivity and permeability; (2) increase of the ratio of permeability to permittivity. In nature, the permittivity of conductive magnetic materials is often much larger than its permeability, and changing the material structure does not reduce the gap between the material permeability and the dielectric constant much. Therefore, in composite materials, it is often the magnetic material that determines the electromagnetic absorption of the composite material. Hence, the first attempt to improve the absorption of the composite material is to choose a magnetic material with excellent magnetic properties.

## 4. Conclusions

Cobalt ferrite was synthesized using a solvothermal method, and the effect of reaction time on phase, structure, electromagnetic properties and microwave absorption of cobalt ferrite was studied. The conclusions of the research are as follows:

(1) The effect of reaction time on the synthesis of cobalt ferrite has been intensively studied. The synthesis of cobalt ferrite hollow spheres is related to the gas-assisted maturation mechanism of Ostwald. While with the increase of reaction time, the spheres are broken and recombined because of the effect of high temperature and electron magnetic field repulsion between magnetic particles. the cobalt ferrite undergoes the process of forming cobalt ferrite, forming hollow spheres, hollow structure damaging, aggregates dispersing and agglomerating again in 4–16 h. Thus it can be seen that cobalt ferrites with different structures can be obtained by reasonable reaction time.

(2) When the reaction time is 8 h, the cobalt ferrite is hollow spheres. On the one hand, the interface polarization relaxation loss is enhanced. on the other hand, when microwave are incident, multiple reflections can be formed in the internal cavity, which improves the attenuation ability of electromagnetic waves, so CoFe_2_O_4_-8h shows better microwave absorption—the effective absorption bandwidth is 9.84 GHz (6–15.84 GHz) for a thickness of 1.72–3.72 mm, and there is a minimum return loss of −47.24 dB at 2.17 mm.

(3) During the preparation of the absorption material, the ratio between the electromagnetic parameters needs to be considered. The absorber with strong strength, wide frequency band and thin thickness can be obtained by adjusting the permittivity and permeability.

## Figures and Tables

**Table 1:** Fitting parameters for the imaginary part of the effective permittivity spectrum.

Sample	*f* (max) (GHz)	*f* (fit) (GHz)
CoFe_2_O_4_-8h	3.92;9.52;12;14.56;18	4.08;9.28;11.92;14.8;17.84
CoFe_2_O_4_-12h	3.52;9.6;12.32;14.88;18	3.68;9.44;12.24;14.8;18
CoFe_2_O_4_-16h	3.76;9.44;11.6;14.96;18	3.68;9.28;11.28;14.86;18

**Table 2:** Fitting parameters for the imaginary part of the effective permeability spectrum.

Sample	*α_i_*	X_0_	*f_ri_/*GHz
CoFe_2_O_4_-8h	0.64; 0.64; 0.63; 0.83	0.194; 0.223; 0.208; 0.222	5.84; 7.36; 9.52; 12.08
CoFe_2_O_4_-12h	0.73; 0.75; 0.72; 0.81	0.258; 0.296; 0.251; 0.221	3.36; 4.64; 6.72; 9.28
CoFe_2_O_4_-16h	0.76; 0.71; 0.69; 0.79	0.226; 0.238; 0.204; 0.195	4.16; 6.08; 8.56; 11.28
